# Dilute acid catalyzed fractionation and sugar production from bamboo shoot shell in γ-valerolactone/water medium†

**DOI:** 10.1039/c8ra02891e

**Published:** 2018-05-14

**Authors:** Qing Qing, Xiaohang Gao, Pengbo Wang, Qi Guo, Zhong Xu, Liqun Wang

**Affiliations:** Department of Biochemical Engineering, College of Pharmaceutical Engineering and Life Science, Changzhou University Changzhou 213164 Jiangsu China wlq@cczu.edu.cn

## Abstract

Overcoming the recalcitrance barrier of cellulosic biomass for efficient production of fermentable sugars at low cost is the current limitation for the industrialization of lignocellulosic biorefineries. In the present work, a two-step non-enzymatic strategy was developed for the fractionation of the main components in bamboo shoot shell (BSS) and conversion of polysaccharides into fermentable sugars by dilute acid in a γ-valerolactone (GVL)/H_2_O solvent system. About 86.0% of lignin and 87.4% of hemicelluloses were removed in the first step by 0.6% H_2_SO_4_ under 140 °C for 1 h with the addition of 60% GVL. The residue solids enriched with cellulose were then subjected to acid hydrolysis employing 0.05% H_2_SO_4_ as the catalyst in 80% GVL at 180 °C for 20 min. The maximum total soluble sugar yield achieved in the acid hydrolysate was 70.7%. This research could provide valuable insights into the valorization of lignocellulosic biomass and become a promising alternative to the biomass-derived carbohydrate production scheme.

## Abstract 2

A non-enzymatic method is reported for the production of fermentable sugars from lignocellulose with a high total sugar recovery.

## Graphical abstract

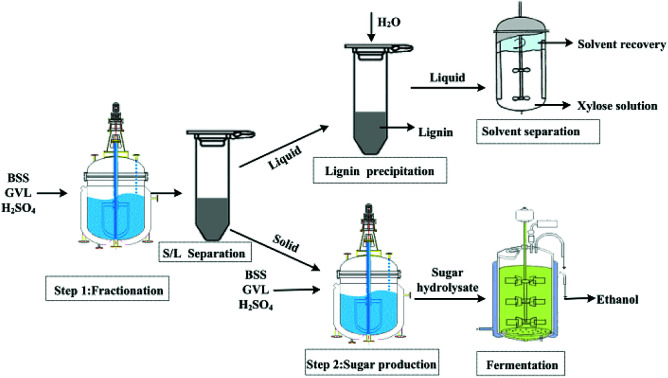

## 1. Introduction

Lignocellulosic materials that could be biologically or chemically converted to fuel, ethanol and other value-added chemicals are abundant in nature.^1^ However, great challenges still remain in how to cost-effectively achieve high conversions of both cellulose and hemicelluloses to fermentable sugars, which is vital to the overall economics of the process.^2^ In general, two different routes are usually taken to fulfill this process, including concentrated acid hydrolysis and enzymatic hydrolysis after the pretreatment of lignocellulose substrates by different techniques.^3^ The former is seriously impeded by corrosive effects, handling hazards and the complexity of acid recovery. The enzymatic hydrolysis method is currently the dominant route, but high enzyme cost is still a concern that prevents commercialization of biofuels.^4^ In this context, great endeavors have been focused on finding novel and economically viable methods to enhance the conversion of cellulose and hemicellulose to monomer sugars amenable for conversion into renewable liquid fuels or value-add chemicals.^5–7^

Lignocellulosic biomass has a complex and rigid cell wall structure which consists of three principal biopolymers, namely cellulose, hemicellulose, and lignin. Cellulose is a long chain polymer composed of glucose linked end-to-end by β-(1,4)glycosidic bonds and forms intra-molecular hydrogen bonds between adjacent chains and itself, whereas hemicelluloses are often dominated by short chains of hetero-1,4-β-d-xylan with degrees of polymerization from 70 to 200 in an amorphous, highly branched structure. Lignin is composed of three different monolignol monomers incorporated into lignin as phenylpropanoids with increasing methoxylation, which are *p*-hydroxyphenyl (H), guaiacyl (G), and syringal (S), respectively.^8,9^ To make the overall process of a lignocellulosic biorefinery economically viable, these three components of lignocelluloses must be effectively recovered and fully utilized.^10,11^

A noteworthy non-enzymatic saccharification strategy employing γ-valerolactone (GVL) as the solvent phase, together with a small portion of water was recently reported to depolymerize cellulose and hemicelluloses to soluble sugars in a flow-through reactor.^12^ GVL has a low melting point (−31 °C), a high boiling point (207 °C) and very low toxicity has been regarded to be a very promising solvent.^13^ It was reported that 70–90% of soluble sugars could be obtained from various feedstock without enzymes by conducting the reaction with a progressive temperature increased from 160 to 220 °C and a GVL/H_2_O ratio of 4 : 1. GVL was found to have an excellent capacity in dissolving lignin and exposing a fresh surface for acid hydrolysis of cellulose and hemicelluloses. However, in order to break the rigid structure of lignocelluloses, high doses of acid catalysts or high reaction temperatures were still required, thus 20–30% of the sugars were easily degraded and formed byproducts, such as furfural (FF), 5-hydroxymethylfurfural (5-HMF), and levulinic acid (LA), with the increasing of the reaction temperature to 220 °C.^14,15^ In addition, a large amount of solvent must be used to wash out the soluble sugars to prevent further degradation in the flow-through reactor, which resulted in product dilution and difficulty in product and solvent recovery. Furthermore, the decomposition products of cellulose, hemicellulose, and lignin were dissolved in the GVL/H_2_O solution as a mixture, which required further separation to make them applicable. In this context, it was speculated that a considerable portion of hemicelluloses and lignin could be removed under a relatively lower reaction temperature since they are more vulnerable to the reaction temperature. Without the protective effects of the hemicelluloses and lignin, the regenerated cellulose residue might be more easily degraded by an acid catalyst under a milder reaction condition to avoid further degradation of monomer sugars to HMF and LA. According to Arrhenius's law, the rate of sugar degradation (*E*_a_ = 130–140 kJ mol^−1^) could be significantly slowed down compared to the rate of cellulose decomposition (*E*_a_ = 90 kJ mol^−1^) when the reaction temperature decreases.^3^ Accordingly, more soluble sugars might be able to recovered in such a modified two-step reaction system.

Bamboo shoot shell (BSS) is a plentiful bioresource in China, which was usually burned directly in the field or discarded, resulting in serious environment pollution and waste of agricultural resources. BSS could be an ideal feedstock for biorefining because it was found to have a large portion of carbohydrate polymers and a low lignin content. To maximize the soluble sugar yields, in this study, we report a two-step non-enzymatic strategy that fractionates the three main components of BSS and recovers cellulose and hemicelluloses as soluble monomers and oligosaccharides in different streams based on an acid catalyzed GVL/H_2_O solvent system. Solid residues rich in cellulose were obtained by fractionation of BSS at mild temperatures (140 °C) with dilute H_2_SO_4_ acid and then subjected to decomposition by acid catalyzed hydrolysis in a GVL/H_2_O medium. A considerable amount of monomer sugars and oligosaccharides were produced in this system which were fermentable to produce ethanol or other liquid fuels. The recovered solids in each step were characterized by FTIR and XRD to provide detailed insights into the chemical and structural alterations by this method.

## 2. Materials and methods

### 2.1. Materials

The BSS used in this study was collected from a farm nearby Changzhou (Jiangsu Province, China) and was washed with deionized (DI) water and dried at 45 °C until it was a constant weight. The dried BSS was milled to a particle size between 20–40 mesh, and was then stored at room temperature for future use.^16^ GVL (98%) and d-glucose (99%) were purchased from Sigma-Aldrich Corp (USA). Concentrated sulfuric acid (95–98%) and phenol (99%) were purchased from Linfeng Chemical Reagent Co. Ltd. (Jiangsu, China) and all other chemicals used in this research were of reagent grade and purchased from Sinopharm Group Chemical Reagent Co. Ltd. (Shanghai, P. R. China).

### 2.2. Fractionation of BSS in GVL/H_2_O system

The BSS was mixed with dilute H_2_SO_4_ and GVL/H_2_O solvent in a high-temperature and high-pressure stainless-steel reactor (Zhenjiang Dantu Universal Electrical Equipment, China) with magnetic stirring. The influences of the operating parameters, such as H_2_SO_4_ concentration (0.2–0.6 w/w%), GVL to water ratio, reaction temperature (120–140 °C), and reaction duration (40–60 min), were evaluated to maximize the removal of hemicelluloses and lignin. After the reaction, the reactor was immediately quenched in an iced water bath and the solid and liquid fractions were then separated by vacuum filtration. After that, fresh DI water was added into the filtrate with a volume ratio of 1 : 10, and part of the dissolved lignin components were precipitated and filtered to separate from the liquid. The solid sample was then dried at 45 °C in a vacuum oven. The collected dry solids were used for further processing and analyses.

The solid recovery, cellulose recovery, delignification, hemicellulose removal and lignin recovery were calculated according to the following equations:
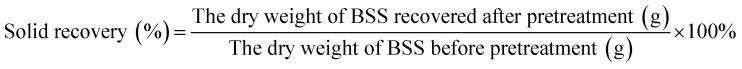

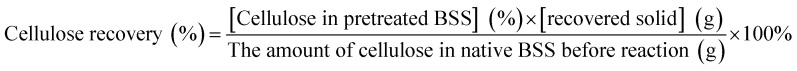

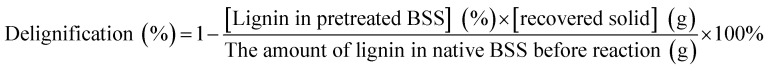

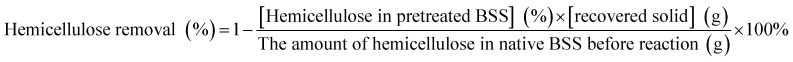

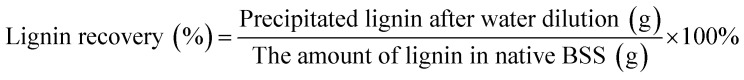


### 2.3. Dilute acid hydrolysis of pretreated BSS

The cellulose solid residues collected from the first step were hydrolyzed using dilute H_2_SO_4_ in a GVL/H_2_O solvent system under various reaction conditions in the same stainless-steel batch reactor. The impacts of H_2_SO_4_ concentration (0.005–0.20 w/w%), GVL to water ratios, reaction time (5–80 min), and pretreatment temperature (160–190 °C) were evaluated to optimize the production of soluble sugars. After the reaction, the liquid was separated from the solid residue and all the sugar and degradation products were further analyzed after dilution to suitable concentrations.

### 2.4. Fermentation of recovered acid hydrolysates

The recovered sugar solution was extracted multiple times using an extractive nonylphenol (NP) and the total sugar concentration was determined to be 20.3 g L^−1^. *Saccharomyces cerevisiae* (Angel Yeast Co., Ltd., China) particles were diluted with sterile water and cultured at 30 °C for 24 h on solid YPD medium (10.0 g L^−1^ yeast extract, 20.0 g L^−1^ peptone, 20.0 g L^−1^d-glucose, 20.0 g L^−1^ agarophyte), after which the cell cultures were harvested and washed with sterile DI water. Washed cells were suspended in 50 mL sterile water, and 0.5 mL was transferred to the hydrolysate with 5 g L^−1^ NH_4_Cl, 1 g L^−1^ MgSO_4_·7H_2_O, and 1 g L^−1^ KH_2_PO_4_. Fermentation was performed under an anaerobic environment in a shaker with a temperature of 30 °C and a rotation speed of 80 rpm. Samples were taken at different time periods to determine the glucose and ethanol concentrations in the fermentation broth. The biomass density (OD_600_) was measured using a UV-vis spectrophotometer, and other concentrations were analyzed using HPLC.

### 2.5. Carbohydrate determination

The chemical compositions of BSS were determined following the National Renewable Energy Laboratory (NREL) analytical procedure (LAP).^17^ 0.3 g of the BSS sample was treated with 72 wt% sulfuric acid (3 mL) in a water bath (30 °C) and stirred with a glass rod once every 10 min for 1 h to recover all polysaccharides as soluble sugars. The H_2_SO_4_ concentration was diluted to 4% by adding 84 mL water, which was then transferred to a sterilized vial and heated to 121 °C for 1 h to further hydrolyze the oligomers to monomers. This second step is sometimes referred to as a post-hydrolysis step, and includes the use of a standard of pure sugar treated at identical conditions to determine the extent of degradation (usually less than 5%).^18^ After the reaction, the vials were cooled to room temperature and an appropriate amount of liquid was taken and the pH adjusted for further analysis. The remaining solids were separated and dried in an oven, which were finally burned in a muffle furnace at 550 °C to determine the lignin and ash content.

### 2.6. Product analysis

The concentrations of monosaccharides (primarily glucose and xylose) together with other soluble carbohydrates and degradation products were quantified using a Waters Alliance HPLC system (Model 2695, Waters Corporation, Milford, MA), employing an Aminex HPX-87H column (Bio-Rad Laboratories, Hercules, CA) and a refractive index detector (Waters 2414). The product yields were calculated as follows:
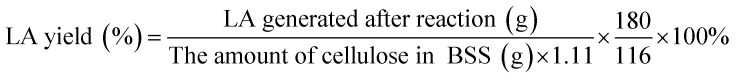

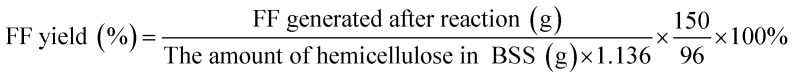

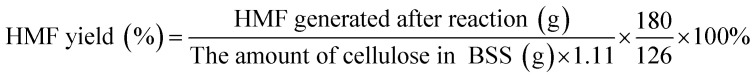

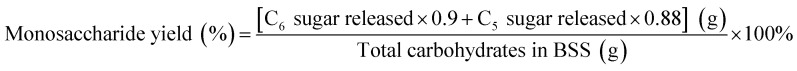


The parameters of 180, 150, 126, and 96 were used to refer to the molecular weight of glucose, xylose, HMF, and furfural. Additionally, 1.136 and 1.11 are the conversion coefficients of xylan to the equivalent amount of xylose and glucan to the equivalent amount of glucose, respectively.

The contents of total soluble sugars, including the monosaccharide and soluble oligosaccharides, were measured using the phenol and sulfuric acid method.^19^ The concentrations of the total soluble sugars were determined using a UV-vis spectrophotometer (Gold S53, Lengguang Tech, P. R. China) and calculated by using d-glucose as a standard according to the absorbance of the saccharification liquids.
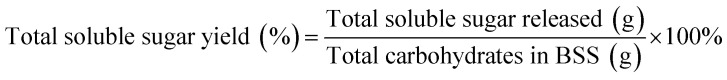


### 2.7. Solid analysis

Fourier transform infrared spectroscopy (FTIR) was performed using a Nicolet PROTÉGÉ 460 FT-IR Spectrometer (Nicolet, USA). Samples were ground and mixed with the spectroscopic grade KBr then pressed into a standard device. The data were recorded in the range of 4000–500 cm^−1^ and the curves were baseline corrected before analysis. Powder X-ray diffractometry (PXRD) was used to examine changes in the crystallinity of BSS solids. PXRD spectra were recorded using a D/max 2500 PC diffractometer with Cu Kα radiation (Rigaku Corporation, Tokyo, Japan). It was operated at a voltage of 60 kV and a current of 300 mA. The curves were recorded in the range of 2*θ* = 5–40° at a step size of 0.02° and a scanning rate of 5.0° min^−1^. The crystallinity index (CrI) was calculated as follows:^20^
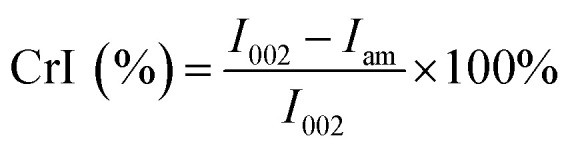
in which *I*_002_ is the intensity of the peak at near 22.1°, and *I*_am_ is the intensity of the peak at near 15.8°.

## 3. Results and discussion

### 3.1. Fractionation of BSS in GVL/H_2_O solution

Based on our preliminary results, the operating parameters of reaction temperature, reaction duration, acid catalyst dosage, and the volume ratio of GVL to water play important roles in the fractionation of lignocellulosic biomass in the GVL/H_2_O solution. As a result, different combinations of these reaction conditions based on an orthogonal experiment design was first investigated to maximize the fractionation of BSS. It has been demonstrated that a pretreatment temperature higher than 140 °C and acid concentration higher than 1% would lead to generation of inhibitory compounds, such as furfural and HMF.^21,22^ Thus, the reaction conditions chosen were comparably moderate to minimize the undesired degradation of sugars. The composition change and solid recovery of pretreated solids were determined as indices for the fractionation performance, while the main component removals were calculated based on the compositional change before and after the reaction. Compared to the untreated BSS, the solid composition was notably changed after reaction for all the tested entries. The solid recovery was decreased with the reaction severity as a result of lignin and hemicelluloses removal. According to the experiment results presented in Table 1, the *R* values for each of the factors were then calculated based on the delignification and hemicelluloses removal of each of the factors and levels. It was found that the concentration of H_2_SO_4_ was the factor that dominated the removal of lignin and hemicelluloses, followed by the reaction temperature. The H_2_SO_4_ acts as a catalyst for both the lignin depolymerization and xylan hydrolysis. All entries (entry 3, 6 and 9) with an acid concentration of 0.6% showed a delignification ratio higher than 70% and a hemicelluloses removal rate higher than 80%. In addition, the dissolution of lignin monomers into the solution was more obviously influenced by the volume ratio of GVL than the reaction duration, while the impact of the GVL ratio on hemicellulose removal was trivial. Among the 12 entries evaluated, entries 9–11 exhibited the best performance, which could meet our requirements for main component fractionation and generation of cellulose rich residues. Moreover, in light of the solid and cellulose recovery after the reaction, the conditions of entry 11 were selected as the most favorable test. The cellulose content of the solids collected from this entry was 83.0%, while the hemicelluloses and lignin were only 11.5% and 4.6%, respectively. The lignin dissolved into the GVL/H_2_O solution was further precipitated by adding water in a ratio of 1 : 10 (v/v). The recovered lignin could be valorized to produce biopolymers and other value-added products.^23^

**Table 1:** Fractionation of BSS in GVL/H_2_O solvent under different reaction conditions

Entry	Reaction condition				Solid recovery (%)	Composition (%)			Delignification (%)	Hemicellulose removal (%)	Lignin recovery (%)
	GVL (%)	H_2_SO_4_ (%)	*T* (°C)	Time (min)		Cellulose	Hemicellulose	Lignin			
0					100.0	36.3 ± 2.7	24.7 ± 1.7	8.8 ± 0.3			
1	40	0.2	120	40	58.5 ± 3.3	50.1 ± 1.6	31.7 ± 2.3	11.6 ± 0.6	22.7	24.8	11.4 ± 0.7
2	40	0.4	130	50	36.0 ± 2.3	70.9 ± 2.4	16.7 ± 1.1	10.4 ± 0.4	57.2	75.7	11.4 ± 0.8
3	40	0.6	140	60	30.0 ± 1.7	81.2 ± 3.3	9.2 ± 0.4	7.6 ± 0.5	73.9	88.8	45.5 ± 2.2
4	60	0.2	130	60	58.5 ± 3.4	52.5 ± 2.6	32.5 ± 2.5	10.9 ± 0.4	27.2	22.9	11.4 ± 1.0
5	60	0.4	140	40	31.0 ± 2.7	82.5 ± 3.1	10.1 ± 0.8	6.4 ± 0.3	77.5	87.3	22.7 ± 1.7
6	60	0.6	120	50	29.5 ± 2.0	80.6 ± 2.9	11.6 ± 0.5	7.2 ± 0.5	75.8	86.2	11.4 ± 0.8
7	80	0.2	140	50	42.8 ± 2.8	52.7 ± 2.6	25.7 ± 1.0	16.4 ± 0.9	20.2	55.4	22.7 ± 1.2
8	80	0.4	120	60	45.5 ± 2.1	59.0 ± 3.5	20.8 ± 1.3	13.7 ± 0.7	29.0	61.7	11.4 ± 0.9
9	80	0.6	130	40	29.0 ± 1.3	83.2 ± 3.2	10.9 ± 0.6	4.8 ± 0.3	84.1	87.2	34.1 ± 2.1
10	60	0.6	140	40	27.5 ± 1.7	80.8 ± 3.7	12.7 ± 0.5	5.6 ± 0.2	82.4	85.8	45.5 ± 3.1
11	60	0.6	140	60	29.5 ± 1.5	83.0 ± 3.1	11.5 ± 0.3	4.6 ± 0.2	86.0	87.4	42.7 ± 1.1
12	60	0.6	130	40	30.5 ± 2.6	80.4 ± 3.6	12.6 ± 0.7	7.0 ± 0.3	75.7	84.4	34.1 ± 1.9

### 3.2. Acid hydrolysis of cellulose residue to soluble sugars

According to Table 1, the main component of the residues collected from the fractionation step was cellulose, and there was only a small portion of hemicelluloses and lignin remaining. Besides the dissolution effects on lignin, GVL was also reported to have dissolution and decrystallization effects on cellulose by affecting the stabilization of the acidic proton relative to the protonated transition states compared to reactions that occurred in H_2_O.^22,24^ Therefore, the recovered solid residues were further subjected to dilute acid hydrolysis in the GVL/H_2_O solution, and various reaction conditions were evaluated to maximize the concentration of soluble sugar in the hydrolysate, as well as to minimize the generation of inhibitory degradation products. The impacts of acid catalyst concentration, reaction temperature, duration, and the volume ratio of GVL to water, were evaluated sequentially.

**Fig. 1:**
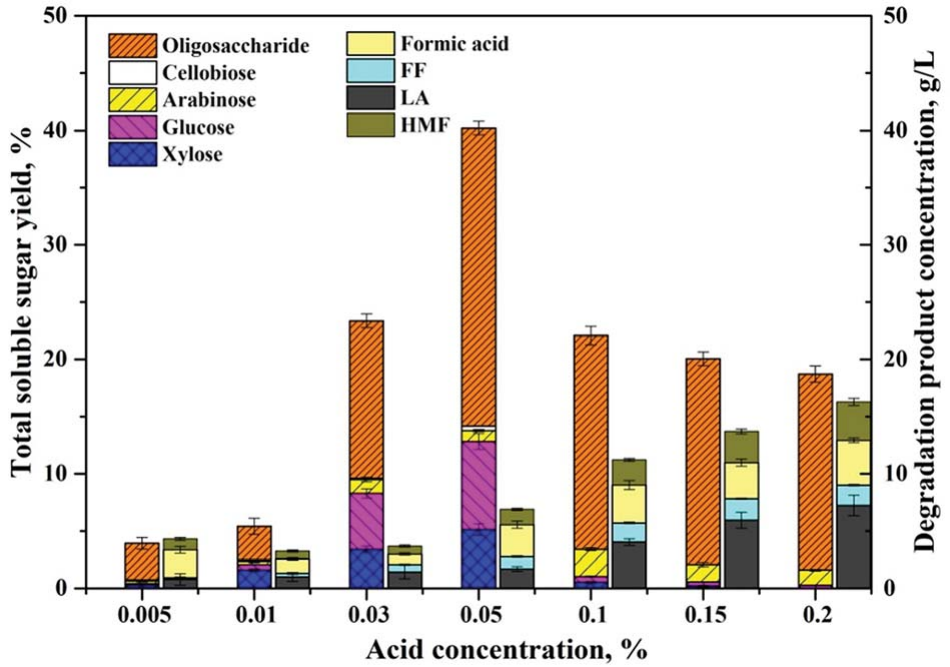
Soluble carbohydrate concentrations produced with different concentrations of H_2_SO_4_ (0.005–0.2%). Reaction conditions: 5 wt% cellulose-rich solid collected after first step, 80 wt% GVL and 20 wt% water, 175 °C for 60 min.

The sulfuric acid applied in the solvent system acts as a catalyst for the cleavage of the β-1,4 linkage. However, it was also known that with the increase of acid concentration, the excessive protons in the hydrolysate might lead to degradation of monomer sugars to furan products.^18^ Therefore, we decided to use an ultra-low acid concentration (<0.2%) for the acid hydrolysis of the cellulose residue to soluble sugars (monomers and soluble oligosaccharides). As shown in Fig. 1, the total soluble sugars, including monomers and oligosaccharides degraded from cellulose and remaining hemicelluloses, were determined under the reaction condition of 175 °C, 60 min, 80% GVL, and with different acid catalyst concentrations from 0.005–0.2%. When the acid concentration was lower than 0.01%, there was almost no soluble sugar detected in the reaction liquor and the solid BSS appeared as a colloid after separation from the solvent, which could be due to the dissolution effect of GVL. When the acid concentration increased to 0.05%, the soluble sugar concentration in the reaction liquor reached the maximum value, and the total yield of glucose and xylose was 12.9%. It was also noticed that the soluble oligosaccharides increased with the enhancement of the acid concentration. However, when the acid concentration exceeded 0.1%, more oligosaccharides were detected than monomers, probably due to the fast degradation of the monomer sugars to furfural, HMF, LA, and formic acid (FA). As implied in Fig. 1, the concentration of the degradation products gradually increased with the addition of acid catalysts. As a result, the acid concentration of 0.05% appears to be the best choice, yielding a total soluble sugar concentration of 40.5% and a degradation product concentration of 7.1 mg mL^−1^.

**Fig. 2:**
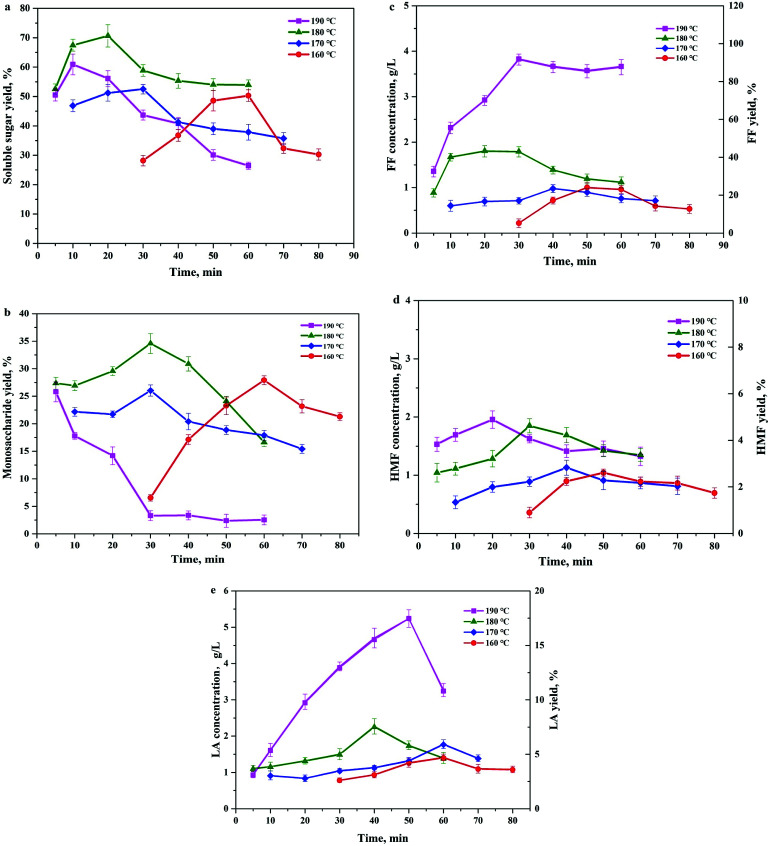
Impacts of reaction temperature (160–190 °C) and duration (5–80 min) on the concentration of total soluble sugars (a), monomer sugars (b), FF (c), HMF (d), and LA (e). Reaction conditions: 5 wt% cellulose-rich solid, 0.05% H_2_SO_4_, 80 wt% GVL and 20 wt% water.

To further optimize the soluble sugar production, reaction temperature and duration were tested within ranges determined by the preliminary experiments. As reflected in Fig. 2, the total soluble sugar yields (Fig. 2a) shows a trend of increasing first and then decreasing as the reaction time progresses. The maximum sugar concentrations were achieved within different time spans, and the required reaction time was shorter under higher reaction temperatures. The optimized soluble sugar yield obtained under the evaluated conditions was 70.7%, which was achieved under the reaction temperature of 180 °C and reaction time of 20 min. However, the maximum monomer sugar yield was detected at 180 °C and 30 min, which was 33.6% based on the total carbohydrate in the BSS (Fig. 2b). When the reaction time exceeded the optimized condition, the soluble sugar and monomer sugar in the hydrolysates diminished simultaneously, indicating a further degradation of monomers and break down of long chain oligosaccharides. Corresponding to the change in the sugar concentrations, the concentration of the degradation products, such as FF, HMF, and LA, also showed a close relationship with the variation of reaction temperature and time (Fig. 2c–e). Due to the low hemicellulose content in the reaction substrate and the fast reaction kinetics at high temperature, the FF yield in the hydrolysate increased significantly (higher than 90%) when the reaction temperature increased to 190 °C. The hexoses in the hydrolysate were further degraded to HMF as the intermediate product. Then the generated HMF was rapidly rehydrated to form LA and FA as the final products in the presence of the acidic catalyst at a relatively high temperature.^25^ Therefore, the HMF concentrations detected in the hydrolysate were considerably low and the decrease of HMF was faster under higher reaction temperatures. The LA yields (Fig. 2e) showed a dramatic increase at the reaction temperature of 190 °C, perhaps due to the high sugar degradation rate at this high temperature according to Arrhenius's law.^3^

**Fig. 3:**
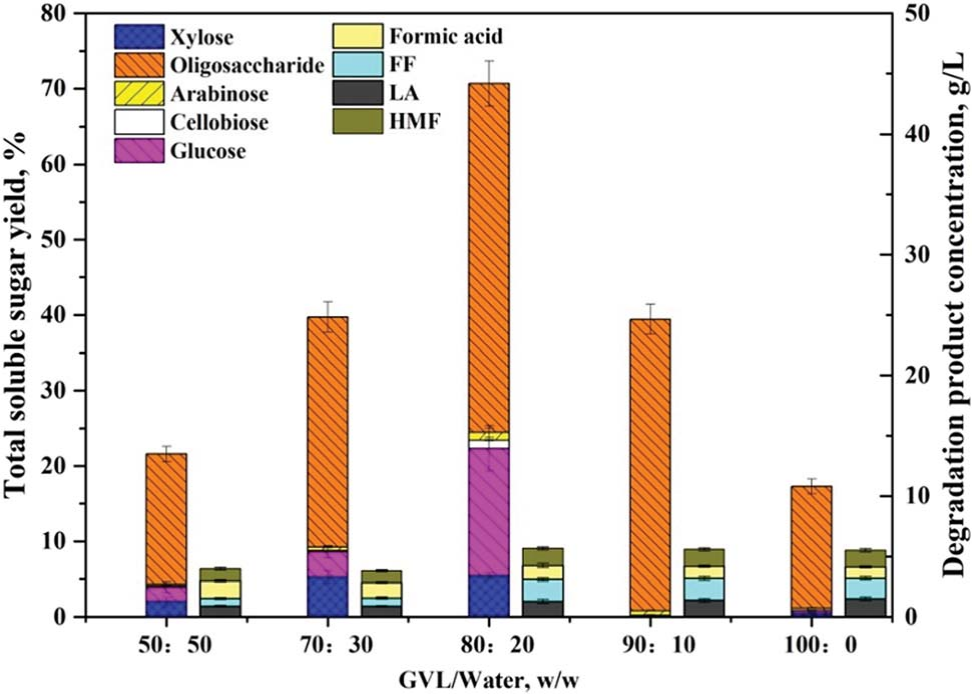
Total yields of soluble carbohydrates in different GVL ratio solvents (50–100 wt%). Reaction conditions: 5 wt% cellulose-rich solid, 0.05% H_2_SO_4_, 180 °C, 20 min.

**Fig. 4:**
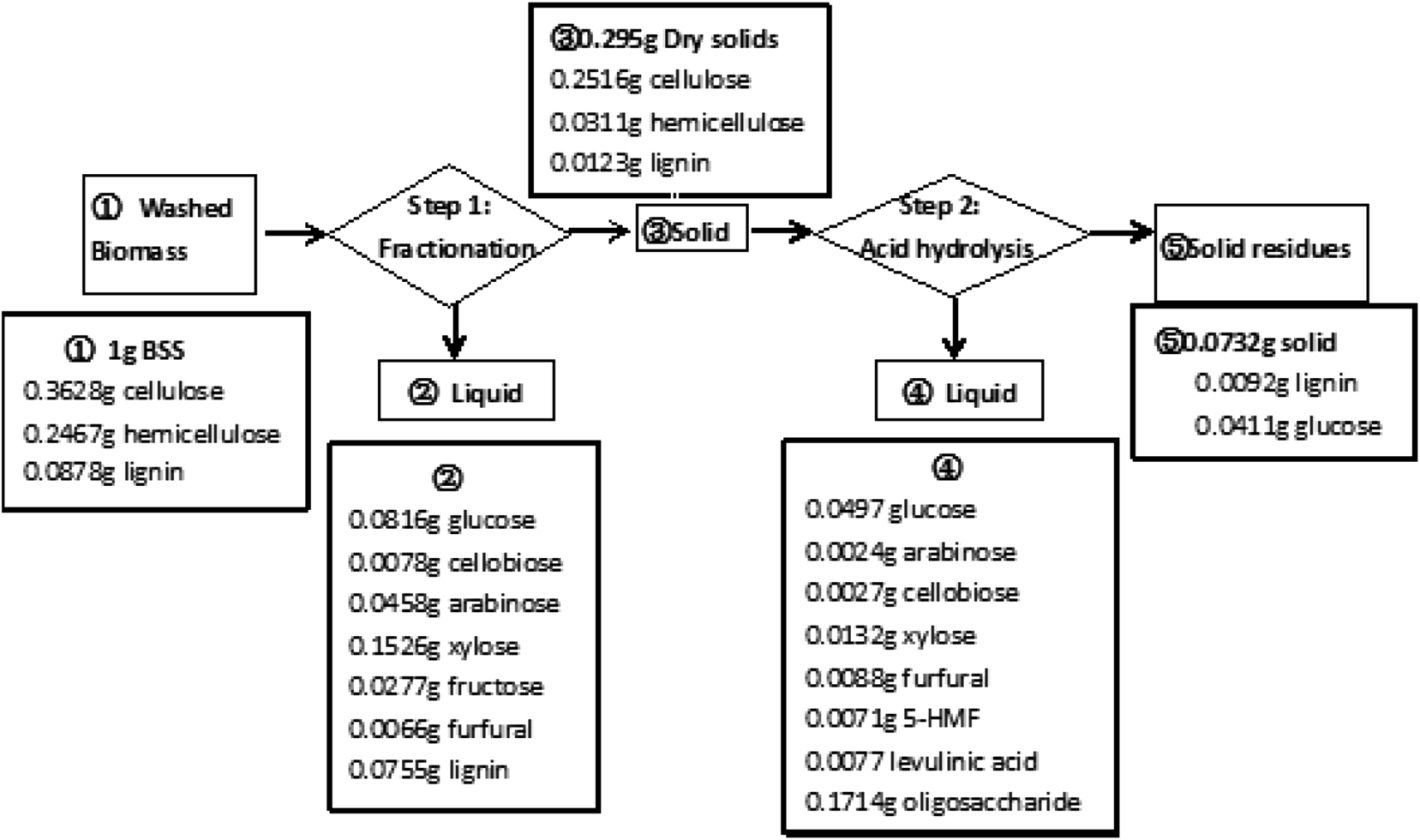
The mass balance analysis of the soluble sugar production scheme by the proposed two-step non-enzymatic method.

A noteworthy phenomenon found was that the ratio of GVL had a profound impact on the total soluble sugar and degradation product yields as shown in Fig. 3. The reaction carried out in the mixture containing 80 wt% of GVL and 20 wt% water (80/20 GVL/H_2_O) resulted in the highest efficiency for the releasing of soluble sugars and monomers (glucose and xylose). Oligomers were important intermediates during cellulose hydrolysis, and their concentration increased with the ratio of the GVL solvent. The generation of degradation products also accreted with the increasing of the solvent ratio, but the concentration of monosaccharides decreased suddenly when the GVL ratio was higher than 80%. As a consequence, the GVL solvent content was determined to be 80% and 70.7% of soluble sugar was achieved under the reaction temperature of 180 °C and reaction time of 20 min by this solvent ratio. In addition to that, the detailed analysis of the material balance was carried out for this two-step non-enzymatic sugar production method as shown in Fig. 4. The most prominent feature of this method is the high total soluble sugar recovery, which was 80.9% (calculated based on the total carbohydrate in the native BSS) if both steps were included.

### 3.3. Fermentability of the recovered acid hydrolysate

**Fig. 5:**
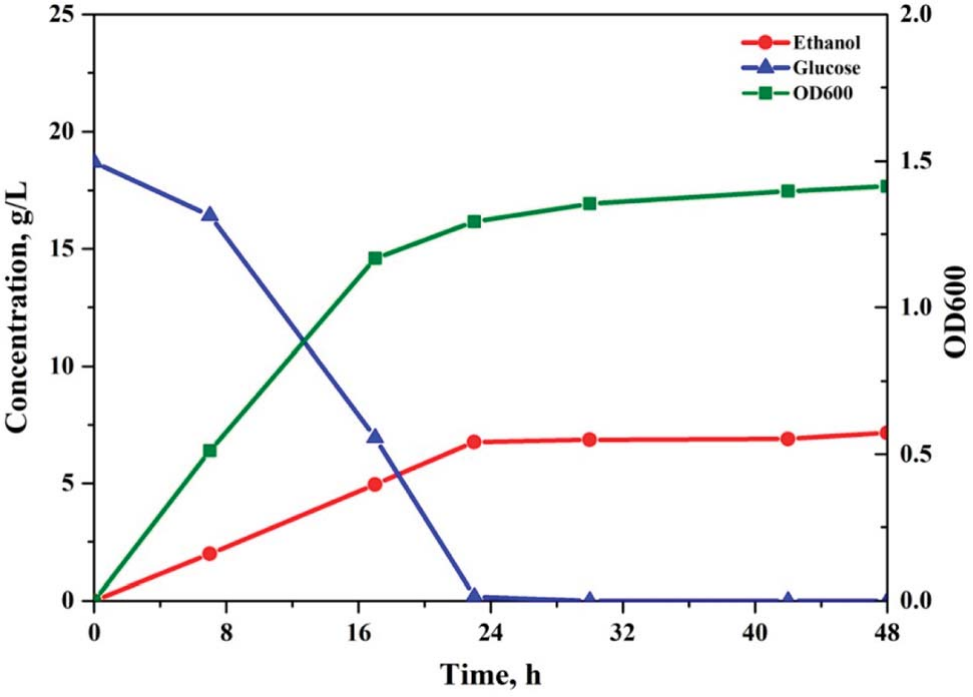
Ethanol fermentation analysis of the soluble sugar produced in the proposed system. Fermentation conditions: 2 wt% *Saccharomyces cerevisiae* strain, 20.3 g L^−1^ of soluble sugars in 80 rpm shaker at 30 °C.

The soluble sugar solution obtained under the optimal reaction conditions was converted to monomer sugars by acidic hydrolysis. The recovered glucose solution was subjected to the fermentation test to evaluate possible inhibitory effects of the degradation products to the fermentation microorganisms (Fig. 5). Most of the GVL was effectively removed by using NP as an extractant.^26^ A hydrolysate containing 20.3 g L^−1^ of soluble sugars (18.7 g L^−1^ glucose and 1.6 g L^−1^ xylose) was obtained. Within 24 h, glucose was consumed completely and the ethanol concentration was 7.2 g L^−1^, which was equivalent to 75.4% of the theoretical yield. This ethanol yield is slightly lower than previously published values that were obtained through fermentation of enzymatic hydrolysate, which might be due to the negative impact of extraction residues in the fermentation broth. Simultaneously, the fermentation results demonstrated that the sugar solution produced by the proposed method did not show significant inhibition effects, and that by using the NP extraction method, most of the GVL could be successfully removed from the hydrolysate. Therefore, the two-step non-enzymatic method could be a promising alternative for the efficient production of fermentable sugars from lignocellulosic biomass.

### 3.4. Structural characterization of solid residues

**Fig. 6:**
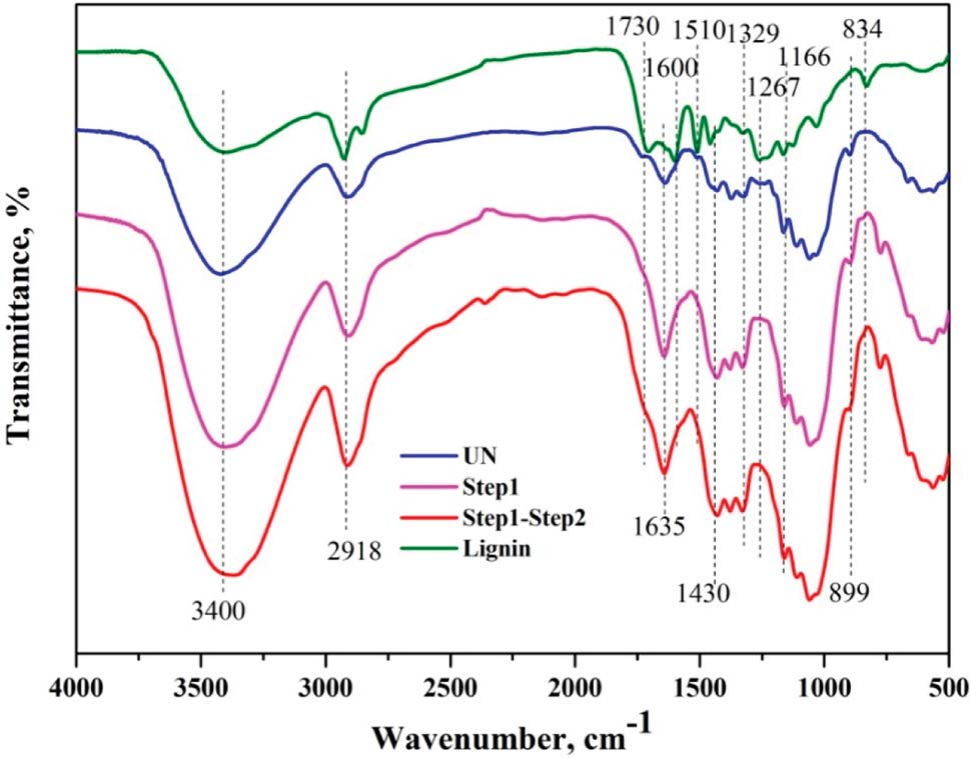
FTIR spectra of the solid residues collected from different steps.

FTIR analyses of the native BSS, solids collected after the first and second steps of reaction, along with the precipitated lignin solid were performed to investigate the chemical changes in each step, as shown in Fig. 6. The wide band at 3400 cm^−1^ was associated with stretching of the O–H hydrogen bonds of cellulose and the band at 2918 cm^−1^ was attributed to the C–H stretching vibrations of cellulose/lignin.^27^ It was evident that the absorption of both peaks was enhanced after each step of the reaction, indicating the increase of cellulose content in the solids. In addition, the absorption of these two peaks in the precipitated lignin sample was considerably weak, while the absorption peaks at 1600 and 1510 cm^−1^, that stands for the aromatic skeletal vibration, were visible. The absorptions at 1329 and 1267 cm^−1^ were attributed to the C–H being out-of-plane in position 2 and 6 of the syringyl type units in lignin, and the band at 834 cm^−1^ corresponded to that in the *p*-hydroxyphenyl units.^28^ The disappearance of the absorption band at 1730 cm^−1^ and the reduction of the band at 1166 cm^−1^ indicated the cleavage of ester bonds from hemicelluloses and lignin after each step. In addition, the band at approximately 899 cm^−1^ was attributed to the β-1,4-glycosidic linkages between the sugar units in cellulose,^29^ and the band at 1430 cm^−1^ was determined to be the HCH and OCH in plane bending in the crystalline region of cellulose, both of which indicated a change of the cellulose portion in the solids.

**Fig. 7:**
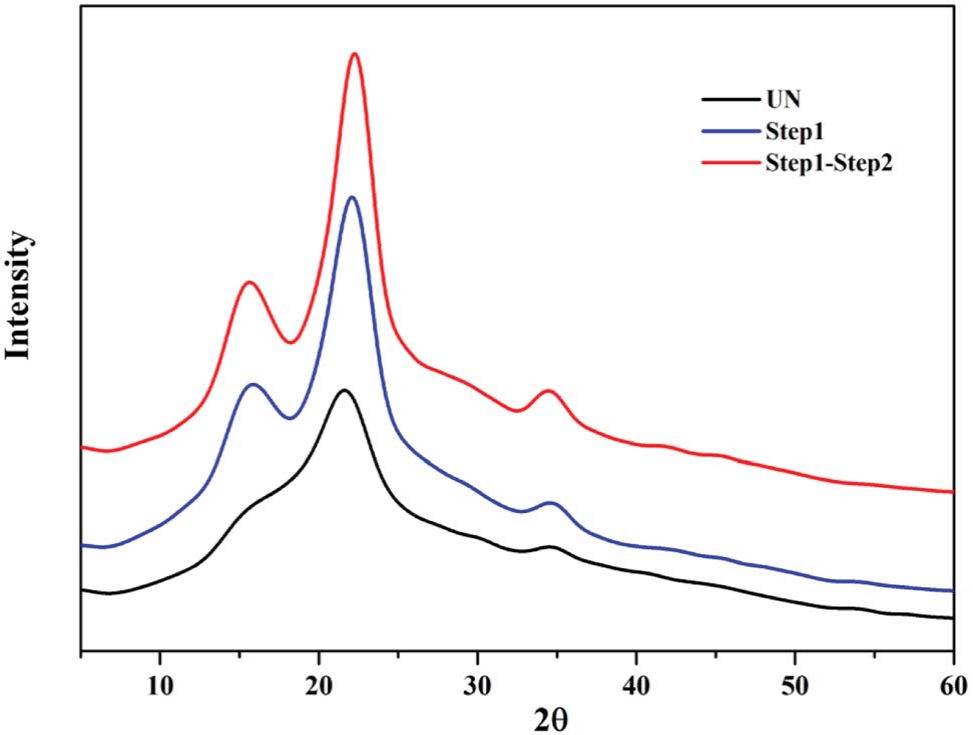
XRD patterns of the BSS solids collected from different steps.

In addition to the changes taking place in the structure, the XRD patterns also provide information on the changes that occurred in the crystalline structure of BSS. The CrI of untreated BSS was 36.4%, which increased up to 46.5% and 52.5% after step 1 and step 2 (Fig. 7). The increment of the CrI indicated a removal of the amorphous components in BSS, such as hemicelluloses, lignin, and part of the amorphous cellulose, resulting in an increased crystalline cellulose proportion that was ready to be converted to soluble sugars in the proposed solvent system.

## 4. Conclusion

A dilute acid catalyzed GVL/H_2_O system was applied to fractionate BSS, resulting in a xylose-rich solution, cellulose solid residue, and precipitated lignin, which were recovered in different streams. The pretreated solids were then hydrolyzed by ultra-diluted H_2_SO_4_ in a GVL/H_2_O solution to produce soluble sugars that are amenable to ethanol fermentation. The total soluble sugar produced was 70.7% based on this two-step non-enzymatic method, indicating the meaningfulness of this method as a competitive alternative to current sugar production scenarios in lignocellulosic biorefinery processes.

## Conflicts of interest

There are no conflicts to declare.

## Supplementary Material

RA-008-C8RA02891E-s001

## References

[cit1] Himmel M. E. (2009). Chemsuschem.

[cit2] Wyman C. E. (2003). Biotechnol. Prog..

[cit3] Shuai L., Questell-Santiago Y. M., Luterbacher J. S. (2016). Green Chem..

[cit4] Klein-Marcuschamer D., Oleskowicz-Popiel P., Simmons B. A., Blanch H. W. (2012). Biotechnol. Bioeng..

[cit5] Tester J. W. , Drake E. M., Driscoll M. J., Golay M. W. and Peters W. A., Sustainable Energy: Choosing Among Options, The MIT Press, 2012

[cit6] Hülsey M. J., Yang H., Yan N. (2018). ACS Sustainable Chem. Eng..

[cit7] Deng W., Wang Y., Yan N. (2016). Current Opinion in Green and Sustainable Chemistry.

[cit8] Boerjan W., Ralph J., Baucher M. (2003). Annu. Rev. Plant Biol..

[cit9] Vanholme R., Demedts B., Morreel K., Ralph J., Boerjan W. (2010). Plant Physiol..

[cit10] Gang C., Xin Z., Simmons B., Singh S. (2015). Energy Environ. Sci..

[cit11] Cao S., Pu Y., Studer M., Wyman C., Ragauskas A. J. (2012). RSC Adv..

[cit12] Luterbacher J. S., Rand J. M., Alonso D. M., Han J., Youngquist J. T., Maravelias C. T., Pfleger B. F., Dumesic J. A. (2014). Science.

[cit13] Horváth I. T. (2008). Green Chem..

[cit14] Mellmer M. A., Sener C., Gallo J. M., Luterbacher J. S., Alonso D. M., Dumesic J. A. (2014). Angew. Chem..

[cit15] Alonso D. M., Bond J. Q., Dumesic J. A. (2010). Green Chem..

[cit16] Qing Q., Zhou L., Huang M., Guo Q., He Y., Wang L., Zhang Y. (2016). Bioresour. Technol..

[cit17] Sluiter A. , Hames B., Ruiz R., Scarlata C., Sluiter J., Templeton D. and Crocker D., Determination of structural carbohydrates and lignin in biomass, 2008

[cit18] Luterbacher J. S., Alonso D. M., Dumesic J. A. (2014). Green Chem..

[cit19] Dubois M., Gilles K. A., Hamilton J. K., Rebers P. A., Smith F. (1956). Anal. Chem..

[cit20] Qing Q., Hu R., He Y. C., Zhang Y., Wang L. Q. (2014). Appl. Microbiol. Biotechnol..

[cit21] Le H. Q. H., Ma Y., Borrega M., Sixta H. (2016). Green Chem..

[cit22] Fang W., Sixta H. (2015). Chemsuschem.

[cit23] Ragauskas A. J., Beckham G. T., Biddy M. J., Chandra R., Chen F., Davis M. F., Davison B. H., Dixon R. A., Gilna P., Keller M. (2014). Science.

[cit24] Mellmer M. A., Alonso D. M., Luterbacher J. S., Gallo J. M. R., Dumesic J. A. (2014). Green Chem..

[cit25] Qing Q., Guo Q., Wang P., Qian H., Gao X., Zhang Y. (2018). Bioresour. Technol..

[cit26] Luterbacher J. S., Alonso D. M., Rand J. M., Questell-Santiago Y. M., Yeap J. H., Pfleger B. F., Dumesic J. A. (2015). Chemsuschem.

[cit27] Chen H., Zhao J., Hu T., Zhao X., Liu D. (2015). Appl. Energy.

[cit28] Zhao X., Dai L., Liu D. (2009). J. Appl. Polym. Sci..

[cit29] Lu Q. L., Tang L. R., Wang S., Huang B., Chen Y. D., Chen X. R. (2014). Biomass Bioenergy.

