# Supplementary material for: Dilute acid catalyzed fractionation and sugar production from bamboo shoot shell in γ-valerolactone/water medium
Source: RSC Adv. 2018 May 14;8(31):17527–34. doi: 10.1039/c8ra02891e (PMC9080427; doi:10.1039/c8ra02891e)
Supplement: RA-008-C8RA02891E-s001 [file RA-008-C8RA02891E-s001.pdf]

Electronic Supplementary Material (ESI) for RSC Advances.
This journal is © The Royal Society of Chemistry 2018

Table S1 Intuitive analysis of the separation of BSS under different conditions.

Entry		Reaction condition				Delignification (%)	Hemicellulose Removal (%)
		GVL (%)	H_2SO_4 (%)	T (°C)	Time (min)		
1		40	0.2	120	40	22.7	24.8
2		40	0.4	130	50	57.2	75.7
3		40	0.6	140	60	73.9	88.8
4		60	0.2	130	60	27.2	22.9
5		60	0.4	140	40	77.5	87.3
6		60	0.6	120	50	75.8	86.2
7		80	0.2	140	50	20.2	55.4
8		80	0.4	120	60	29.0	61.7
9		80	0.6	130	40	84.1	87.2
10		60	0.6	140	40	82.4	85.8
11		60	0.6	140	60	86.0	87.4
12		60	0.6	130	40	75.7	84.4
Delignification	Mean 1	51.3	23.4	42.5	61.4		
	Mean 2	60.2	54.6	56.2	51.1		
	Mean 3	44.4	77.9	57.2	43.4		
	Range	15.7	54.6	14.7	18.1		
Hemicellulose Removal	Mean 1	63.1	34.4	57.6	66.4		
	Mean 2	65.5	74.9	61.9	72.4		
	Mean 3	68.1	87.4	77.2	57.8		
	Range	5.0	53.0	19.6	14.6		
